# Standardization of Procedures to Contain Cost and Reduce Variability of Care After the Pandemic

**DOI:** 10.3389/fsurg.2021.695341

**Published:** 2021-06-24

**Authors:** Federico Raveglia, Riccardo Orlandi, Arianna Rimessi, Fabrizio Minervini, Ugo Cioffi, Matilde De Simone, Angelo Guttadauro, Marco Scarci

**Affiliations:** ^1^Thoracic Surgery, San Gerardo Hospital, Azienda Socio Sanitaria Territoriale (ASST)-Monza, Monza, Italy; ^2^Thoracic Surgery, Lucerne Cantonal Hospital, University of Lucerne, Lucerne, Switzerland; ^3^Department of Surgery, University of Milan, Milan, Italy; ^4^Department of Surgery, Istituti Clinici Zucchi, University of Milan Bicocca, Monza, Italy

**Keywords:** thoracic surgery, lean, pandemic (COVID-19), lobectomy (lung), sigma six, lean six sigma

## Abstract

The coronavirus disease 2019 (COVID-19) pandemic has changed many aspects of our private and professional routine. In particular, the lockdowns have severely affected the entire healthcare system and hospital activities, forcing it to rethink the protocols in force. We suggest that this scenario, in spite of the new challenges involving so far complex healthcare providers, may lead to the unique opportunity to rethink pathways and management of patients. Indeed, having to resume institutional activity after a long interruption that has completely canceled the previously existing schemes, healthcare providers have the unique opportunity to overcome obsolete and “we have always done in this way” model on the wave of the general desire to resume a normal life. Furthermore, the pandemic has highlighted some flaws in our health system, highlighting those critical issues that most need to be addressed. This article is a review of pre-pandemic literature addressing the use of Lean Six Sigma (LSS) and standardization processes in thoracic surgery to improve efficiency. Our goal is to identify the main issues that could be successfully improved along the entire pathway of a patient from the first referral to diagnosis, hospitalization, and surgical operation up to convalescence. Furthermore, we aim to identify the standardization processes that have been implemented to achieve significant improvements in patient outcomes while reducing costs. The methods and goals that could be used in the near future to modernize our healthcare systems are drawn up from a careful reading and interpretation in light of the pandemic of the most significant review articles in the literature.

## Introduction

The coronavirus disease 2019 (COVID-19) pandemic has changed many aspects of our private and professional routine. In many countries, patients were confined to their homes and access to healthcare facilities for the non-COVID cases was not so easy as most hospital facilities shifted their focus from the care of acute patients to patients with COVID. The lockdowns have severely affected the entire healthcare system, forcing it to rethink the protocols in force. This has led to dramatic changes in surgical practice as well, in particular, thoracic surgery. At the beginning of the pandemic, the main issues were the following: how to offer surgery to patients with cancer considering that (1) surgery requires a lot of human and healthcare resources that were depleted being redeployed elsewhere, (2) patients with lung cancer are at high risk of mortality with COVID-19 and are especially vulnerable after surgery, and (3) patients undergoing surgery require clinic visits, laboratory and imaging tests, and an inpatient stay that will result in a considerable number of personal contact points.

Thoracic surgeons addressed these points with an enormous effort by (1) introducing new guidelines for triaging operations (1) and (2) redistributing resources (2).

In spring 2020, European countries ended their lockdowns and healthcare systems resumed their operations while being faced with new challenges. During the first wave, the pressing issue was to select the most urgent cases, and later on, the issues were the following: to (1) restart cancer screening programs, (2) increase outpatient appointments to cope with the backlog of visits, (3) reconstitute specialist teams that were dismantled, and (4) continue to ensure the safety of patients throughout their journey at the hospital as the virus still persisted in the community. Unfortunately, the second wave of the pandemic has hit us again, further destabilizing a so far volatile situation.

We suggest that this scenario may lead to the unique opportunity to rethink pathways and management of patients. Indeed, healthcare providers have the unique opportunity to overcome obsolete models on the wave of the general desire to resume a normal life.

This article is a review of pre-pandemic literature addressing the use of Lean Six Sigma (LSS) and standardization processes in thoracic surgery to improve efficiency. Our goal is to identify the main issues that could be successfully improved along the entire pathway of a patient from the first referral to diagnosis, hospitalization, and surgical operation up to convalescence. Furthermore, we aim to identify the standardization processes that have been implemented to achieve significant improvements in outcomes while reducing costs. Attention was given to those that were severely affected by the pandemic.

The first issue that we would like to tackle is the reduction in the length of stay. Through standardization of protocols, all stakeholders should be aimed at eliminating non-value-added (NVA) steps allowing more efficient rates of the day of surgery admission (DOSA). As described earlier by Sofela et al. (3), we experienced an incredibly low rate of same-day admissions as well, and in spite of many attempts and great use of financial resources, they thus remained until we applied a systematic approach. Then, we found out the problem that the admitting nursing staff could not cope with the volume of patients and had very little time to do all the prescribed interventions, and the interesting thing though was that nobody even knew that they were doing things like diabetes swap and TED-stocking measurements, which were carried out only the previous week by another team. Removing the NVA steps significantly boosted the same-day admission rates and generated important savings. Healthcare professionals (HCPs) are often stuck in the “we have always done it this way” view and find it difficult to challenge the dogma. A good strategy, in this scenario, is to map the journey of the patients with a post that stuck on the wall and then ask all stakeholders to mark with a different color marker the following: what adds value, what does not, and what is neutral. Further to this exercise, the next step is to remove all NVA steps, discuss the neutral steps one by one, and harmonize the process for those steps that adds value.

The second topic concerns improvements in the waiting time performance in diagnostic assessment of patients with lung cancer. Through standardization of procedures and “waste” elimination, reorganization should be aimed to reduce the time from consultation to diagnosis of lung cancer and improve the experience of care of patients. A good example of this could be the use of government or even self-determined standard of care (SoC) (i.e., first consultation within 2 weeks from referral, scans carried out in another 2 weeks, surgery in 1 month, etc.). It is also very important to collect the opinion of the patients; we recommend the wide adoption of patient-reported outcome measures questionnaires (PROMs) as the only tool to significantly monitor the quality of care.

The third topic concerns saving the cost of and decreasing the risk of surgical procedures, in particular, video-assisted thoracoscopic surgery (VATS) lobectomy. By avoiding customized protocols for individual surgeons, redesigning the process should be aimed to reduce the length of surgical operation, inefficient care transitions, and overuse of resources.

## Lean Six Sigma

**Figure 1:**
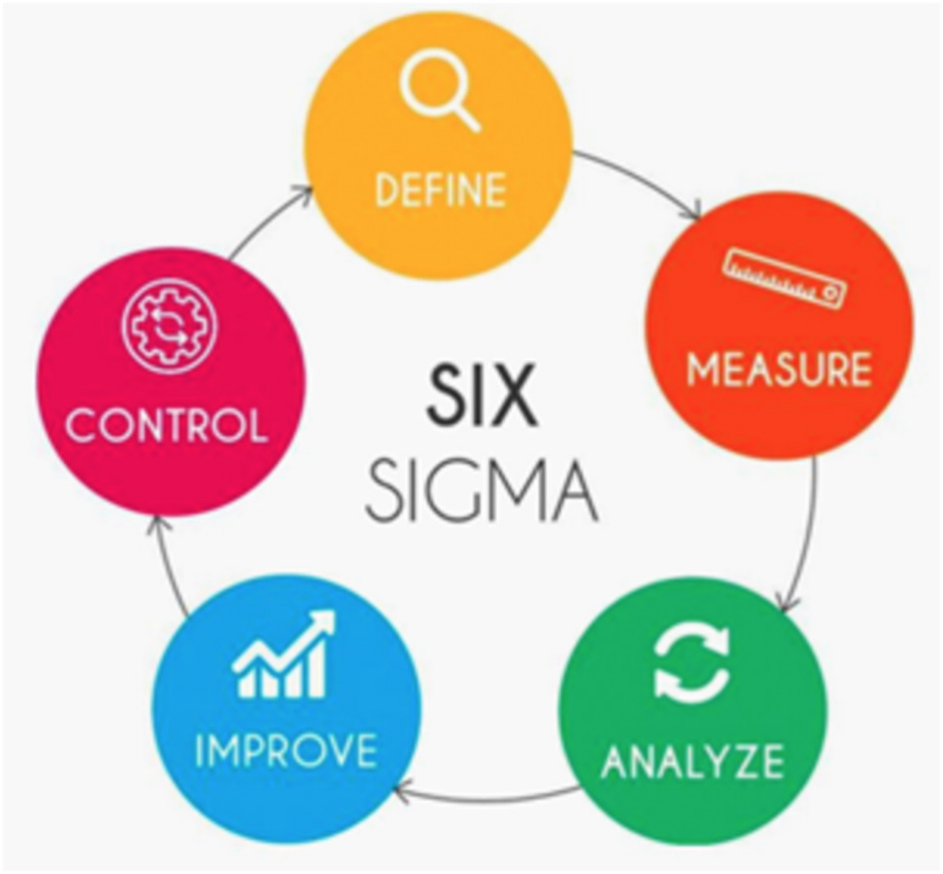
Phases of the six sigma method. Image source: https://www.sweetprocess.com/lean-six-sigma/.

**Figure 2:**
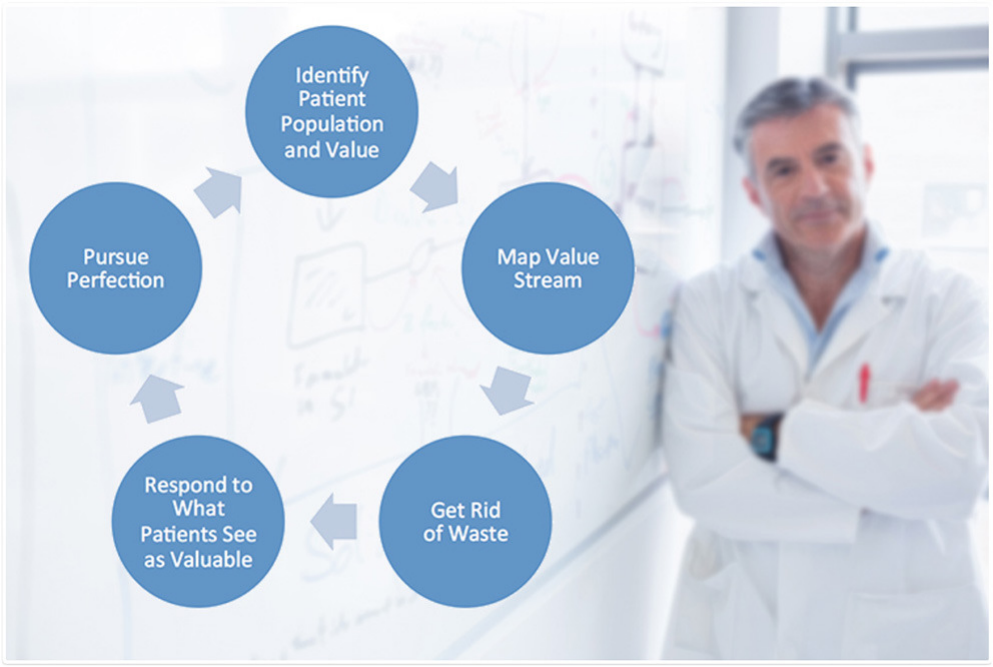
Flow chart showing an example of Lean Six Sigma (LSS) methods applied to medical practice. Image source: https://www.ausmed.com/articles/lean-healthcare.

Lean Six Sigma (LSS) is a method originally used to improve the capability of business processes; it combines lean manufacturing/lean enterprise and relies on a collaborative team effort to improve outcomes by systematically removing waste and variations (Figure 1). LSS methodology has been introduced in healthcare since the early 2000s (4), and its professional framework may be the cornerstone to address some points of post-pandemic uncertainty, and enhance understanding and provide practitioners in the field with effective guidance to manage the COVID-19 crisis and its aftermath (Figure 2). LSS has often been driven by managers or outside consultants hired to fix what was thought to be an acute problem by administrators (5). This is, in our opinion, a very treacherous course of action as all changes to be effective and long-lasting must be driven from the involved workforce in a bottom-up approach and must not be imposed in a top-down approach. In the latter case, HCPs will feel that they have no responsibility and that administrators want to compromise care to save money. Involving HCPs showing what is in it for them and what are the improvements they can achieve in their professional life and care of the patients will, instead, determine a powerful response. Once there is a buying in from some early adopters, most of the others will follow and when they take ownership of projects they will not want them to fail. Interestingly, financial incentives work only for a very short period of time before the situation reverts to the previous condition, showing that to drive changes we need to leverage on deeper motivators such as general and professional well-being. Following these approaches, costs will be reduced pleasing administrators, but cost saving should be a by-product of meaningful staff engagement and service delivery.

## General Improvements for Hospital Stay Decrease in Thoracic Surgery

The ideal program for thoracic patients should be customized to deliver an optimal care experience from referral to discharge. Such a pathway should be primarily aimed to improve outcomes and then to reduce costs. Outcome improvement concerns not only a decrease in postoperative complications but also a reduction in length of stays, fast recovery to baseline activity, and reduction in preoperative and perioperative time as previously described in enhanced recovery after surgery (ERAS) principles. The favorable impact of these issues on money saving is commonly accepted (6, 7).

Brown et al. (8) established a program to decrease the length of stay in hospital after elective thoracic surgery adopting LSS methods. Their project was a single study center involving a multidisciplinary team (MDT) including different professional figures as thoracic surgeons, nurse managers, physiotherapists, patient flow certified nurse midwife (CNM), and radiology administrators.

According to LSS principles, the strategy adopted to improve DOSA was based on identifying and removing the NVA steps. To put it in a nutshell, their project was articulated in four steps that authors called as follows: define, measure, analyze, and improve.

The first consisted in (1) general criteria for identification of patient enrollment, (2) selection of all stakeholders, and (3) creation of a value-stream mapping (VAM) to have an overview of journey state of patients (“AS IS”) from out-patient office to the access to operatory room (OR).

The second consisted in the measurement of data describing the usual practice during the patient pathway and the related opinion on this practice by stakeholders. Data included surgeons, procedure, attendance to preoperative assessment clinic, and DOSA and confirmed the surgical date provided and whether preoperative tests had been completed. On the other hand, opinions of patients and nurses related to the process were obtained and recorded by the paper survey.

The third consisted in data analysis to identify some NVA issues such as the elevated number of steps along the patient pathway, a large volume of rework, and other topics.

The fourth step was the interventive one that consisted of the development and implementation of a presurgical checklist and the weekly planning meeting.

The project lasted 6 months and then authors published their results showing a significant reduction in DOSA quantified with a DOSA>75%. Remarkably, every previous project with the same objective, but without adopting LSS methods, had failed the goal.

Authors identified as major successful factors are the following: the cooperation among different professional figures and the ability of this method in involving people prompting communication and collaboration thanks to a system of the weekly planned meeting.

Procedural standardization is another model adopted to improve the efficiency and safety of surgical tasks and, in particular, thoracic surgery. The standardization process by definition is an activity focused on achieving an optimal degree of order in a determined context concerning actual or potential items and provisions for common and repeated use. Standardization consists of formulating, issuing, and improving standards.

Iwasaki et al. (9) published their experience in the development of a multidisciplinary and multicenter standardization program. Their background was that surgery has become an extremely complex procedure requiring a high level of knowledge and competence by different stakeholders: surgeons and OR nurses above all. So, they started a project on standardization among thoracic surgery departments from different hospitals, with the aim to improve high-quality procedures. Once again, the project was based on joining meetings and collecting data by survey; (1) the survey allowed the collection of data regarding predetermined tasks concerning the perioperative pathway of patients; (2) meetings allowed the discussion among MDT in finalizing the standard for every task. After the introduction of standardization, the authors found that in most of the enrolled centers median operative time and perioperative time decreased, as well as blood loss. They concluded that projects on standardization of specific tasks in thoracic surgery may lead to a significant improvement in quality and at the same time implement participation among stakeholders.

## Improvements of Waiting Time in Diagnosis of Lung Cancer

It is a common opinion that any delay in diagnosis or in the period of time running between suspicious finding and treatment of lung cancer may reduce overall and cancer-specific survival. On this basis, many departments have developed a diagnostic assessment program (DAP) in order to boost this key point in the pathway of patients.

Cotton et al. published their experience with lean six sigma to improve waiting time performance in diagnostic assessment for these patients (10). In particular, they aimed to reach the threshold of 65% of all patients diagnosed within 28 days from referral. The program was established with the help of external consultants experienced in lean six sigma improvement event.

They started with a 4-days Kaizen workshop including different stakeholders (oncologists, nurses, clerical staff, patients and family members, and office and administrative staff) focused on the development of a unit optimization plan. Adopting lean six sigma methodology with the help of the consultants, they were able to draw a VAM of the current and ideal patient pathway, which allowed the identification of waste, rework, bad coordination, and unneeded processes.

The program improved rapidity in diagnosis with a rise from 45 to 75% of cases in the target time and allowed an increase in volumes of patients as well. The review paper is interesting since their conclusion shows how the lean six sigma model was successful in achieving their initial goal. Moreover, it emerges that the use of multidisciplinary cross-programs to improve quality could be adopted in other issues concerning healthcare, which deserve waste elimination. The review paper confirms that the strength of lean six sigma workflow analysis methodology relies on putting together different stakeholders ranging from patients and their families to surgeons, as reported earlier by Morgan et al. (11).

## Cost Saving and Risk Decreasing of Thoracic Surgery Procedures

The intraoperative period is the most meaningful moment along the pathway of patients in a surgical department, since it is the one where the clinical outcome is influenced most and also the one where most of the money is spent. Moreover, this is probably the activity where surgeons are more involved and that has more possibility to change. So, intraoperative time is a high-priority target for efficiency improvement and some authors have oriented their efforts in this field.

Cerfolio et al. published their experience with a project that aimed to reduce the pre-incision time in the OR for lobectomy that adopts the process of lean six sigma (12). Their background was that time spent in the OR by surgeons is mostly represented by non-operating activities and that the cost of every minute in the OR ranges between 22 and US$133. Therefore, they aimed to eliminate waste and, in particular, that activities that have been routinely consolidated over the years without evidence. At the same time, efficiency in terms of clinical outcomes should have been preserved. They embraced a value streaming process and labeled every step of the pathway of patients in the OR according to criteria of necessity; if a step was considered necessary, it added value, otherwise it did not. They were able to delete many procedures. Results confirmed that eliminating arterial catheters, axillary rolls, arm boards, beanbags, central catheters, epidural catheters, and Foley catheters pre-incision time, but also mortality and morbidity, significantly decreased. Once again, the lean six sigma process and VAM have successfully improved clinical practice.

Based on this experience, Kyle et al. in collaboration with Cerfolio, focused their attention on the standardization of surgical instruments with the aim to investigate cost saving (13). They adopted the process of lean six sigma removing all instruments that in their opinion did not add value. Costs were calculated as money spent for instrument purchase, replacement, and processing. This project has led to a cost saving of about US$55 for each VATS procedure. Once again, the use of lean six sigma and standardization of surgical trays allowed to reduce the number of instruments and to decrease costs without adverse effect on clinical outcomes. Farrokhi et al. (14), Guzman et al. in the same year (15), and Stockert (16) have already reached similar conclusions and also underlined how OR turnover has been fastened by lighter trays. Interestingly, they also showed how few instruments on trays lead to a decrease in errors during operation.

Yeo et al. (17) focused on VATS lobectomy with the aim to improve safety and outcomes and to decrease costs in accordance with the value-based healthcare (VBHC) criteria. Their background was the evidence of many wastes in patients undergoing VATS lobectomy at their department.

They performed a review of perioperative procedures for VATS lobectomies and designed a process improvement map to determine the procedures that determined value. Then, they identified sources of high cost and practice variability. The project was developed by introducing standardized practices focused on quality and cost, by establishing a database to record safety and quality and a regular MDT meeting with surgeons, mid-level providers, and clinical staff to evaluate outcomes. The authors showed an overall 187% decrease of time in the OR, significant reduction in chest x-ray, laboratory tests, consultations, global costs, and length of stay.

Finally, Fong et al. published a systematic review of 36 studies focused on improvement in intraoperative efficiency (18) concluding that despite papers reviewed used different approaches and were hardly comparable, evidence was in favor of standardization as a method to improve consistency and speed of workflow by repetition. Moreover, the authors underlined that whatever project has been adopted there were always some common elements to determine success. These were (1) correct data collection, (2) communication among all stakeholders and teamwork, and (3) overall care in patient pathway with special focus on the OR but without forgetting preoperative and postoperative period. In our experience, we have faced several times with significant cost differences for the same operation among the surgeons of the same unit. Any attempt to find a compromise was met with resistance as all surgeons believed that their technique was the best. So we allowed all surgeons in the division to use whatever they wanted, but their results and costs would be disclosed at hospital audit meetings. After 6 months of this exercise, all surgeons aligned procedures and costs to show that, sometimes, peer pressure is very useful to drive meaningful changes.

## Discussion

Severe acute respiratory syndrome coronavirus 2 (SARS-CoV-2) pandemic has severely decreased volumes of thoracic surgery procedures since hospitals canceled most of the non-urgent interventions. Therefore, a progressive increase in deferred cases during the pandemic is expected and will require completion within a short period once the pandemic has subsided. However, if only pre-pandemic capacity will be available, then poor operating capacity and patient management could cause substantial delays and increase morbidity and mortality.

Hospitals and departments must focus their efforts to increase operating capacity, by rethinking the protocols in force, in order to quickly clear the backlog of deferred cases. In this manner, we will be able to address the emergency and at the same time to improve our models for the upcoming future. In doing so, the pandemic will be also an opportunity. However, the key point is how to drive innovation. With an eye on pre-pandemic experience, we have reported how adopting change driving methods, such as the LSS and standardization, improvement of quality, cost saving, and favorable clinical outcomes, are achievable. We advocate the use of LSS by HCPs using a bottom-up approach, with managers acting as facilitators rather than decision-makers. The pandemic situation has offered, in our opinion, the possibility of focusing on the whole pathway in a holistic fashion, rather than been forced to accept compromises dictated by local power struggles or customs.

## Author Contributions

FR designed and wrote the review. RO, AR, FM, UC, MD, AG, and MS contributed to review the paper. All authors contributed to the article and approved the submitted version.

## Conflict of Interest

The authors declare that the research was conducted in the absence of any commercial or financial relationships that could be construed as a potential conflict of interest.
